# DAT1 Gene Methylation as an Epigenetic Biomarker in Attention Deficit Hyperactivity Disorder: A Commentary

**DOI:** 10.3389/fgene.2020.00444

**Published:** 2020-05-11

**Authors:** Erika Tonelli, Esterina Pascale, Miriam Troianiello, Claudio D'Addario, Walter Adriani

**Affiliations:** ^1^Faculty of Psychology, Università Telematica Internazionale “Uninettuno”, Rome, Italy; ^2^Medico-Surgical Sciences and Biotechnologies Department, “Sapienza” University of Rome, Rome, Italy; ^3^Clinical Center “TROPOS” for Child Neuropsychiatry, Frascati, Italy; ^4^Faculty of Bioscience and Technology for Food, Agriculture and Environment, University of Teramo, Teramo, Italy; ^5^Center for Behavioural Sciences and Mental Health, Istituto Superiore di Sanità, Rome, Italy

**Keywords:** DNA opposite strand, dopamine transporter, CpG epigenetic marker, CGAS, conners' scales

## Introduction

Attention Deficit/Hyperactivity Disorder (ADHD) is the most common neurodevelopmental alteration in childhood (Curatolo et al., 2009; Purper-Ouakil et al., 2011) characterized by pervasive and impairing symptoms of inattention, hyperactivity, and impulsivity, which often lead to poor academic performance and impaired social interactions (American Psychiatric Association, 2000). The estimated world prevalence for ADHD is around 5% (Polanczyk et al., 2007), with an onset during childhood of symptoms that often persist into adolescence and adulthood (Biederman et al., 2006).

Although the etiology of ADHD is multi-factorial, this syndrome is viewed as a motivational dysfunction. Several neuroimaging and neurophysiological studies have shown a delay in the normal brain development and functional abnormalities in brain areas related to executive functions (Shaw et al., 2013; Hart et al., 2014; Rubia et al., 2014), reporting evidence of imbalanced prefrontal and/or striatal levels of neurotransmitters, especially dopamine (Oades, 1998; Sagvolden and Sergeant, 1998) and altered cross-talk between fronto-striatal circuits (Chambers and Potenza, 2003).

Twin, family and adoption studies have supported a strong genetic base for the disorder, with heritability ranging from 60 to 90% (Goodman and Stevenson, 1989; Biederman et al., 1990; Levy et al., 1997). Pre-, peri-, and postnatal environmental factors play an important role. Maternal lifestyle during pregnancy and parent-infant interactions have been proposed to affect the phenomenological expression, resulting in a more, or less severe constellation of symptoms (Milberger et al., 1996; Coffin et al., 2005; Neuman et al., 2007).

For the identification of genes involved in ADHD, several studies have been conducted (Li et al., 2006; Wood and Neale, 2010), but to date no determinant genetic marker has been identified. ADHD candidate genes have been hypothesized, including dopamine and serotonin transporters and the dopamine D4 and D5 receptors (Klein et al., 2017).

## DAT1 Gene and Its Epigenetic Modulation

Recent research has focused on the dopamine transporter (DAT) because modifications in the expression and/or function of this gene may well lead to ADHD symptoms (Bannon, 2005; Jucaite et al., 2005). Furthermore, subjects with ADHD respond well to drugs that inhibit DAT, including methylphenidate. DAT1 polymorphisms have also been associated with other psychiatric disorders like generalized anxiety, social phobia, obsessive-compulsive disorder, and Tourette's (Rowe et al., 1998; Gadow et al., 2008).

DAT1 gene has a 40 bp variable number tandem repeat (VNTR) in the 3'-untranslated region, which exist in 3–13 copies; the 9–10-repetition alleles are the most common. Although not all literature agrees, the 10-R allele has often been implicated in the presentation of ADHD (Cook et al., 1995; Gill et al., 1997; Waldman et al., 1998; Curran et al., 2001).

Epigenetic study supports a pivotal role in neuronal development, differentiation, cell communication, and synaptic plasticity (Levenson and Sweatt, 2006). Among epigenetic mechanisms, the methylation of DNA (leading to gene silencing) is the best characterized and studied. DNA methylation mostly occurs in the promoter CpG islands, where the bases converted to 5-methylcytosine, directly exert negative effects on the expression of genes. DNA methylation has been recently implicated in the development of psychiatric disorders, such as bipolar disorder, depression, and schizophrenia (D'Addario et al., 2012, 2013, 2017). Despite the abnormal methylation of promoter-specific CpG residues could be considered as a stable signature in complex psychiatric disorders, the association between DNA methylation level and a given disease is quite inconsistent. For instance, both increased and unchanged level of methylation were highlighted in patients with schizophrenia (Bromberg et al., 2008; Carrard et al., 2011).

## DAT1 Methylation Analysis—Previous Data

Recently, in order to highlight objective biomarkers useful for the diagnosis of ADHD, some research has focused on DNA methylation in the promoter of DAT1 gene. Xu et al. (2015) examined the association of such epigenetic marker with ADHD among Chinese Han children. In his study, which compared 50 ADHD patients with 50 non-ADHD control subjects, a prominent decrease was shown in DAT1 and other dopaminergic genes DRD4 and DRD5, highlighting a severely restricted dopaminergic system in children with ADHD. In order to verify whether the alterations in DAT1 expression were caused by CpG methylation, Xu et al. searched for a CpG island located in the 5'-UTR, a gene regulation area, identifying 19 CpG sites. Subsequently, the methylation status of these sites was analyzed using bisulfite sequencing. Their results reveal three individual CpG sites (site 7, 8, and 18 in authors' numeration) that showed a significant difference in methylation compared to the control group.

We (Adriani et al., 2018) similarly assessed the epigenetic status of the 5'-UTR region of DAT1 gene. We recruited school-aged children (6–12 years old) diagnosed by routine anamnestic and cognitive evaluation, plus Conners' scales and k-SADS. None of them was a smoker. In general, altered methylation levels were found for six selected CpG sites, for ADHD patients compared to healthy controls. To support this hypothesis, we analyzed the correlation between the subjects' clinical scores and methylation data. We found that hyper-methylation in CpG M1 correlated negatively with CGAS (Children's Global Assessment Scale) value, and slightly with some of the Conners' subscales, thus serving an index of severity for ADHD. Whereas, hyper-methylation in CpG M6 correlated with change of CGAS and Conners' after 6 weeks of therapy, thus serving an index of likelihood for therapeutic efficacy. Interestingly the CpG M5 turned out to be correlated with M6 on one side, and with M1, M2, M3 on the other hand (see also Lambacher et al., 2020).

**Figure 1:**
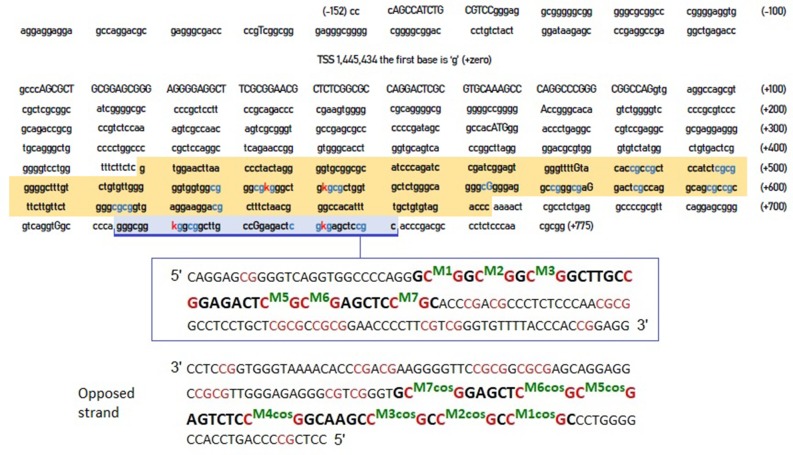
DNA sequence of DAT gene 5'-UTR. The sequence highlighted in orange is that examined by Xu et al. (2015). The area highlighted in blue is the one examined in our previous study (Adriani et al., 2018). The blue CpG islands are those that are likely to be methylated, whereas the red “K” are the specific cytosines that the two studies report to be hyper methylated in ADHD. The inset (below) provides a further detail of the blue-highlighted area, together with its complementary sequence on the opposite strand (reported as forward strand, from its 5' to its 3') and CpG numerations. COS, complementary on the opposite strand.

However, the crucial question was whether the CpG residues we identified were more relevant compared to these identified by Xu et al. (2015). Figure 1 shows the entire DNA sequence of the 5'-UTR region of the DAT, highlighting the areas examined by Xu et al. (2015) and by our previous study (Adriani et al., 2018).

Studies on the DAT1 gene have also been conducted in the research of biomarkers for Tourette syndrome (Müller-Vahl et al., 2017) highlighting an altered DAT methylation in the most severely affected patients. However, the DNA sequence analyzed in this study is before TSS (where mRNA starts) thus not including the 5'-UTR.

## A New Contributor:Opposite- Strand Methylation Level

It should be considered that the fidelity of maintenance of CpG methylation within cell division has been found to be very high in hemi-methylated DNA, on the other hand *de novo* methylation resulted to be quite low (Riggs et al., 1998). Dnmt3a and Dnmt3b methylate DNA *de novo*, and this occurs without regard to the methylation status of the complementary CpG position (Okano et al., 1998). It should also be considered that within each CpG dyad, several enzymes (i.e., DNMT-TET-TDG) could theoretically control as many as 21 cytosine modification states not necessarily in symmetric form (Wu and Zhang, 2014). We thus thought of relevance the evaluation of the reverse strand in order to monitor a possible differential methylation in the two strands in ADHD subjects. For this reason, extending the work previously done (Adriani et al., 2018), we run a new assay for methylation levels on the other strand, which is just facing the previously assessed strand: in other words, we thought of analyzing methylation levels of CpG residues, which are exact *complementary on opposite strand* (“cos”) to the previous, already assessed ones.

We selected 14 ADHD patients (half 9\10 and half 10\10 genotype) for which we assessed the other strand (Figure 1). Once new data available, we performed correlations on methylation levels, expressed as percentage of methylated CpG in the sample: we correlated the newly assessed CpGs (termed from M7-cos to M1-cos) among them and also between all them and old CpGs. The results are shown on Table 1. The M5-cos is correlated with M6-cos whereas the M1-cos, M2-cos, and M3-cos are all strongly correlated one to each other: the profile on the opposite strand is identical to that found for the gene strand (see Lambacher et al., 2020). Moreover, the M6-cos is negatively correlated to M6 while the M1-cos is negatively correlated to M2. The M7 and the M7-cos, although some results were evident, are not taken here in consideration as the original data only point to M1, M2, and M6; therefore, we focused on the functional motifs CGGCGGCGG (M1–M3) and CGCG (M5 plus M6).

**Table 1:** Correlation among the newly assessed CpGs and between all the new CpGs vs. all the old CpGs.

	***CpG***	***CpG***	***CpG***	***CpG***	***CpG***	***CpG***	***CpG***
	***M7-cos***	***M6-cos***	***M5-cos***	***M4-cos***	***M3-cos***	***M2-cos***	***M1-cos***
co M6-cos	−0.071						
co M5-cos	−0.018	0.709					
co M4-cos	0.15	0.525	0.799				
co M3-cos	0.013	0.382	0.397	0.351			
co M2-cos	−0.075	0.307	0.43	0.527	0.599		
co M1-cos	−0.032	0.467	0.356	0.191	0.71	0.691	
co M7	−0.174	−0.198	−0.274	−0.157	−0.1	−0.31	−0.384
co M6	−0.18	−0.421	−0.145	0.006	0.184	0.053	−0.249
co M5	−0.274	0.045	0.014	0.086	0.225	0.083	−0.082
**(M4) N/A**							
co M3	−0.561	−0.066	−0.03	−0.053	0.01	−0.031	−0.131
co M2	−0.159	0.11	0.162	0.173	−0.302	−0.018	−0.447
co M1	−0.03	0.045	−0.151	−0.034	0.177	0.289	0.08

*Pink highlights denote correlation values higher than 0.6411. Green highlights denote the newly discovered negative correlation*.

*The M1-cos, M2-cos, and M3-cos are all strongly correlated one to each other. The M5-cos is correlated only with M6-cos*.

*The M6-cos is negatively correlated to M6 while the M1-cos is negatively correlated to M2*.

*M2 and M1-cos are also physically close on the DNA: a given protein may interact with both, well before the two DNA strands are opened for mRNA transcription*.

*Data, given one candidate cytosine, point to the leftward opposed cytosine (facing the preceding guanine in a motif on that same strand)*.

*It is suited as well to serve a biomarker if compared with the direct opposed cytosine (rightward, facing the following guanine)*.

These new data suggest that not only M1, M2, and M6 located on the same strand of the gene are important: also the M1-cos and M6-cos located on the opposed strand are crucial. While increased M1 (or M2\M6) methylation on the strand of the gene is associated with ADHD (or its relief), very intriguingly the same sites tend to be demethylated on the opposed strand. This is the first time (at least to our knowledge) that the importance of the opposed strand is claimed as important for the role of a given gene within a given pathology. Whereby a CpG residue is claimed important when its methylation is increased, the corresponding CpG residue on the opposed strand might be claimed important when its methylation is conversely decreased.

## A New Approach to CPG Studies

We thought that, if crucial CpG positions exist, these shall be ON (Methylated) on one (gene) strand and moreover OFF (Demethylated) on the other (opposed) strand. The quantity of CpGs in any DNA trait is 1\16 of total, based on simple probability: it is unlikely that all of them are equally crucial. We propose that the identification of a crucial CpG, to cause a given phenotype, may exploit its negative correlation with its complementary opposite.

For first motif CGGCGGCGG the best candidates are M1 and M2; for second motif CGCG the best candidate is M6. By doing this kind of reasoning, we realized that the relevance of M2 and M1-cos, suggested by their being highly correlated (see Table 1), is supported by their being also physically close on the DNA. The cytosine of M2 is indeed close to the cytosine of M1-cos (more than cytosine of M2-cos is with cytosine of M1). As such, where two or more CpGs form a motif, we postulate the following possibility for a functional interaction: given one candidate cytosine (e.g., CpG 2), the leftward opposed cytosine (i.e., CpG 1-cos), facing the guanine of the CpG just preceding the candidate one on that same strand, is as well-suited if compared with the directly opposed cytosine (i.e., CpG 2-cos), which is anyway rightward since it's facing the guanine following the candidate cytosine. Following similar reasoning, the use of M6 and M5-cos should be suggested: despite the fact that these two are not correlated (see Table 1), yet they are physically close on the DNA. The cytosine of M6 is indeed very close to the cytosine of M5-cos (more than cytosine of M6-cos is with cytosine of M6 itself, perhaps).

In order to construct an index, to be then used for clinical purposes, two or more parameters may well be multiplied if they co-vary with the pathology in the same direction. For instance we proposed to multiply methylation with level of DAT-directed auto-antibody (Adriani et al., 2018). Given negative correlations between strands, corresponding residues on the other (cos) strand could be used as well, but they anti-covary: therefore, they could be considered as contributing for the proportion of demethylation. Therefore, the index should multiply the methylation level for gene-strand CpGs with ≪**100 – methylation**≫ for any CpG-cos (see Lambacher et al., 2020).

## Conclusion and Future Perspectives

To date, clinical diagnosis of ADHD is solely based on structured interviews or on questionnaires; as such, risk of subjectivity in the interpretation of outcomes may give rise to doubts about their diagnostic reliability. Previous studies have shown that the determination of DNA methylation in specific CpG residues within the 5'-UTR region of the DAT1 gene can be used as reliable indicator of ADHD (Giana et al., 2015; Adriani et al., 2018). Also, research showed the role of conventional epigenetic components in ADHD, such as MeCP2, histone transferacetylases and de-acetylases. Few critical methylated CpG sites in the DRD4 promoter also exhibit a considerably different pattern in ADHD children, compared to healthy controls (Xu et al., 2015; Dadds et al., 2016).

The purpose of this commentary was to provide an overview about a new possible approach, when searching an epigenetic biomarker for ADHD. Our data presented here suggest indeed a new approach to DNA methylation analysis. Our hypothesis, accordingly, is that the methylation levels on the strand opposed to the candidate gene can be used to identify which CpG sites, on the gene-strand, are really crucial; this, may strengthen their correlations with clinical data.

This is the first time, to our knowledge, that the importance of the opposed strand is claimed. We are suggesting that patterns of methylation on the strand of the gene, specifically for CpG sites M1, M2, and M6 associated with ADHD, establish an unsuspected relationship with patterns of decreased methylation on the opposed strand. This hypothesis clearly needs more studies, which should further assess the level of methylation in many more of the opposite CpG residues. The main limit of studies on ADHD biomarkers is the relatively small size of clinical samples, suggesting to deepen the research with further studies on larger samples. More in general, we are suggesting to evaluate this complementary-strand aspect in all next studies on DNA methylation.

## Author Contributions

WA, EP, and CD'A conceived the study. MT, EP, and CD'A realized the study. ET wrote a first draft with supervision by WA.

## Conflict of Interest

WA, EP and CD'A hold following patent applications: WA, Laviola G, EP, CD'A—“*Metodo per determinare il deficit di attenzione con iperattivitá*” (Method to determine Attention Deficit and Hyperactivity Disorder). Patent Application in ITALY at no. 102016000129938 (22-December-2016); European Patent Application at no. 17830021.6 (21-December-2017). Granstrem O, WA, Laviola G, Porfirio MC, Curatolo P—“*Biomarkers for validation of ADHD (Attention Deficit and Hyperactivity Disorder) diagnosis and monitoring of therapy efficacy*”. Full patent PN810701WO, Int. Application PCT/EP2013/066845, Publication International Number WO/2014/023852 (10-August-2013). The remaining authors declare that the research was conducted in the absence of any commercial or financial relationships that could be construed as a potential conflict of interest.
